# Performance of a simple chromatin-rich segmentation algorithm in quantifying basal cell carcinoma from histology images

**DOI:** 10.1186/1756-0500-5-35

**Published:** 2012-01-17

**Authors:** Kyle Lesack, Christopher Naugler

**Affiliations:** 1 Room G503, O'Brien Centre for the BHSc, 3330 Hospital Drive N.W., Calgary, Alberta T2N 4N1, Canada; 2 Department of Pathology and Laboratory Medicine, University of Calgary and Calgary Laboratory Services, C414, Diagnostic and Scientific Centre 9, 3535 Research Road NW, Calgary, AB T2L 2 K8, Canada

## Abstract

**Background:**

The use of digital imaging and algorithm-assisted identification of regions of interest is revolutionizing the practice of anatomic pathology. Currently automated methods for extracting the tumour regions in basal cell carcinomas are lacking. In this manuscript a colour-deconvolution based tumour extraction algorithm is presented.

**Findings:**

Haematoxylin and eosin stained basal cell carcinoma histology slides were digitized and analyzed using the open source image analysis program ImageJ. The pixels belonging to tumours were identified by the algorithm, and the performance of the algorithm was evaluated by comparing the pixels identified as malignant with a manually determined dataset.

The algorithm achieved superior results with the nodular tumour subtype. Pre-processing using colour deconvolution resulted in a slight decrease in sensitivity, but a significant increase in specificity. The overall sensitivity and specificity of the algorithm was 91.0% and 86.4% respectively, resulting in a positive predictive value of 63.3% and a negative predictive value of 94.2%

**Conclusions:**

The proposed image analysis algorithm demonstrates the feasibility of automatically extracting tumour regions from digitized basal cell carcinoma histology slides. The proposed algorithm may be adaptable to other stain combinations and tumour types.

## Background

The interpretation of digital histology images by pathologists (so called 'tele-pathology') is revolutionizing the practice of anatomic pathology [1,2]. A natural extension of this use of digital images in histology interpretation is the addition of digital analysis tools to aid in diagnosis or the completion of time-consuming tasks. A prime example of the success of this approach is the utilization of algorithm-assisted identification of abnormal cells in cytology preparations [3].

In terms of histology, a number of studies have recently looked at image classification algorithms. One recent use of automated image analysis and processing has been as a part of algorithms used to classify breast cancer tissue. Using a supervised learning method, Petushi et al. developed an algorithm capable of classifying breast cancer carcinomas based on histological tissue micro-texture and spatial position [4]. Using the commercial software packages Matlab, and LNKnet, the algorithm classified the micro-tissue types as nuclear, extra-cellular, or adipose. The algorithm further classified the nucleus into three separate types, each representing a different nuclear morphology. Similarly, an algorithm developed by Karaçali and Tözeren was used to classify breast tissue images based upon tissue texture and spatial distribution [5]. This algorithm was used to classify the tissue images based on the quantity of chromatin and collagen, in addition to a measure of the tissue's spatial heterogeneity. Another breast cancer image analysis algorithm was developed by Hall et al. [6]. This algorithm was developed to assess human epidermal growth factor receptor 2 (HER2) expression in breast cancer tissue. The team used the open source image processing software ImageJ to separate the diaminobenzidine and haematoxylin stains from each other. This was followed by the extraction of the membrane regions from the digitized breast cancer slides. The HER2 score generated using this method was based upon the extracted membrane pixels. Other automated image analyses include oral epithelial dysplasia and squamous cell carcinoma [7], and melanoma [8-11]. However, the application of these pattern recognition algorithms involves complex programming and may serve to assist only in narrow scopes of diagnostic practice.

The aim of our study is to define the operational characteristics (sensitivity and specificity) of a simple colour-based segmentation algorithm for quantifying basal cell carcinoma from photomicrographs. The basis of this algorithm is the observation from anatomic pathology practice that cells with dense chromatin (including many cancer cells) have a different colour spectrum than surrounding normal tissues. Our hypothesis was that the operational characteristics would differ among common basal cell carcinoma subtypes (superficial, nodular and infiltrative) with the subtypes exhibiting more compact chromatin (superficial and nodular) demonstrating better operational characteristics than the infiltrative subtypes.

Basal cell carcinoma was chosen to examine this question as this cancer presents with a well-defined range of histological subtypes and occurs in association with non-neoplastic chromatin-rich cells present in the epidermis and dermis. Finally, because basal cell carcinomas are the most common malignant neoplasm in humans [12], access to clinical material was not a limiting factor. Although basal cell carcinomas are highly curable by surgical intervention, their sheer number (over one million new cases per year in the United States [13]) translates into a heavy burden for health care systems. The only previous study that explored the automated analysis of BCCs was performed by Gutierez et al. [14]. By modelling the visual recognition process, the algorithm used a supervised learning approach to identify regions of interest (ROI). The ROIs identified by the algorithm were found to coincide highly with those selected manually by a pathologist.

### Image analysis overview

In order to extract and analyse features of a digital image, it is first necessary to identify and separate the ROIs. Image segmentation involves dividing an image into regions of similar characteristics based on features such as brightness or morphology [15]. Ideally the foreground of the resulting image contains the desired regions. A simple technique for image segmentation involves segmenting grayscale images based on their pixel intensities [16]. By filtering out pixels above or below a certain threshold value, grayscale images may be segmented into regions of similar brightness. The resulting segmentation can be stored as a new image containing only the black and white values that correspond to the foreground/background regions. More complex thresholding methods are also available. These include the use of multiple thresholds, as well as adaptive thresholding, where the local threshold values are determined according to their neighbouring regions [17]. Further segmentation methods also exist, including seed growing, and boundary based techniques [18]. Prior to feature extraction and analysis, further processing may be required once the image has been segmented. For example, disconnected regions of images may be filled in using morphological operations. This may be accomplished by performing a binary closing operation. Another common operation is noise reduction, frequently achieved by applying mean or median filters [19].

The slides evaluated with this algorithm were stained with haematoxylin and eosin (H&E). Although H&E stains are easily distinguished visually by colour, digitally separating regions containing stain co-localisation is difficult. Separation via colour deconvolution provides a means of separating stains with overlapping regions. The basis of this method is to separate the component stains by performing an ortho-normal transformation of the image's RGB information [20]. Several recent studies have used stain separation by colour deconvolution prior to analyzing cancerous tissue [21-23].

## Methods

**Figure 1:**
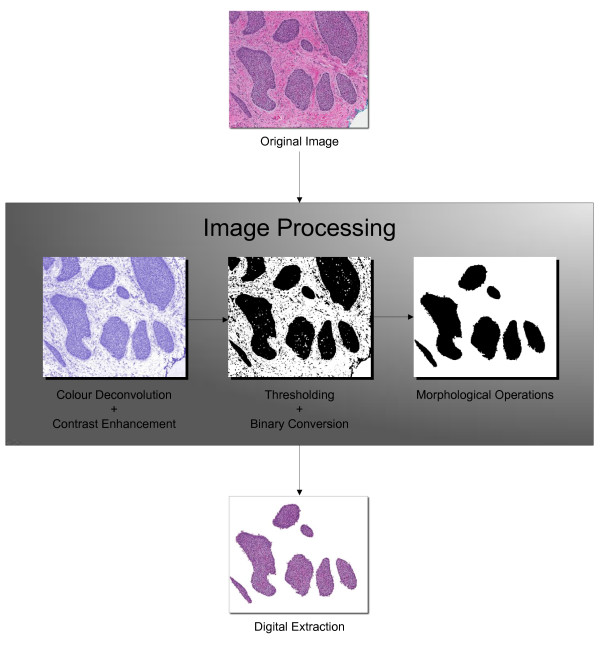
**Overview of the tumour extraction algorithm**.

The algorithm used to extract the BCC tumours consisted of four steps: pre-processing, segmentation, morphological operations, and feature extraction (see Figure 1). A copy of the algorithm used is available as a macro in Additional file 1. The macro also provides the specific parameters used in the algorithm. During pre-processing, colour deconvolution was used to separate the haematoxylin stain from each of the images. The resulting image was then segmented based upon pixel intensities. Subsequently, morphological operations were performed to connect the discontinuous regions that resulted from the segmentation process. Finally, area-based particle analysis was used to extract and quantify the ROIs from the image. This analysis allowed the performance of the algorithm to be evaluated.

### Case selection/image acquisition

Cases were selected from a convenience sample of basal cell carcinomas reported by the senior author as part of his clinical sign-out practice. Digital images of 30 H&E stained BCC histology slides were obtained using a commercial Aperio CS-O slide scanner at 80 × magnification. Sections containing BCC were stored using the JPEG format (1072 × 902 pixels).

### Software

The open source image processing and analysis program ImageJ was used in this study. First released in 1997 by software developer Wayne Rastban, ImageJ is an open source program based on the National Institutes of Health's NIH Image. Current features consist of numerous image processing and analysis operations, including image segmentation and extraction, noise reduction, image transformations, and particle analysis. These features are further expanded upon by an active user base. There are currently hundreds of downloadable user plugins and macros [24]. Additional benefits of this software include the support of numerous file formats, and platform independence [25]. As a result of being platform independent, ImageJ is capable of running on multiple operating systems, including MS Windows, Apple OS, and Linux. The algorithm described below was used in conjunction with version 1.44 of ImageJ. With the exception of the colour deconvolution plugin, all of the processes performed are available using the default ImageJ commands.

### Digital image processing and analysis

#### Colour deconvolution

The colour deconvolution plugin by Gabriel Landini [26] was used to separate the BCC images into separate images containing the haematoxylin and eosin stain components using the built-in H&E vector. The plugin creates an additional image corresponding to the complement of the haematoxylin and eosin stains. Because the chromatin-rich basophilic (nuclear) regions were of interest, only the 8-bit Haematoxylin images were retained. The colour deconvolution process was followed by contrast enhancement in order to facilitate the segmentation process.

#### Segmentation

Thresholding was then used to segment the pixels darker than the threshold value. The ImageJ isodata algorithm [27] was used along with the automatic thresholding option. This algorithm This process resulted in a binary file containing only black and white pixels, where the black pixels corresponded to the regions above the threshold value.

#### Morphological operations

Due to the lack of intense haematoxylin staining in the non-basaloid cell regions, the binary images produced during the segmentation process frequently contained holes and disconnected regions in the tumour nests. As a result, morphological operations were performed on the segmented images. Hole filling was achieved using a combination of median filtering and binary closing operations. Initially a median filter was applied to the bright outliers using the ImageJ *Remove Outliers *command. This was followed by a binary closing operation, and median filtering of the dark outliers.

#### Feature extraction

As other baseloid and chromatin-rich features (e.g. single lymphocytes, hematoxylin stain precipitates, microcalcifications, etc.) could produce false positive results, we attempted to remove these features through a filtering step using the ImageJ particle analyzer feature. A minimum particle size of 750 pixels was used in order to exclude non-tumour nest particles. The extracted tumour was then obtained by removing all particles outside of the ROIs.

#### Analysis

The evaluation of a given algorithm is inherently subjective and biased towards the author's preferences, as standard methods for evaluating the algorithm do not exist [28]. For the purpose of this analysis a manual evaluation of tumor nests was used as the ground truth dataset.

To accomplish this, one of us (CN) manually evaluated printed photomicrographs of the 30 basal cell carcinoma images: 10 each of nodular, infiltrative and superficial subtypes. For each of these images, all tumour nests present were manually delineated with a black marker, scanned and analyzed with a manual approach. The main challenges in evaluating an extraction algorithm are determining the true dataset (ground truth), and the appropriate performance metrics [29,30].

A further challenge is the lack of standardized image extraction algorithms, seeing that most existing algorithms are optimized for a specific task. This causes a further problem for evaluating the algorithm, and the colour deconvolution approach in particular. In order to assess the effect of using colour deconvolution, the same set of histology slides were analyzed using grayscale based thresholding in place of the colour deconvolution step. In the comparison algorithm, the image was first converted to an 8-bit grayscale image, and the colour deconvolution step was omitted. The remaining steps were carried out as described by the proposed algorithm.

The binary images of the algorithmically extracted tumour nests were subtracted from the binary images obtained by manual evaluation. The resulting image, containing the areas of the image not extracted by the algorithm, was considered to contain only false negative (FN) pixels. Similarly, the binary images of the manually extracted tumours were subtracted from the algorithmically extracted ones. The resulting image quantified the pixels considered to be false positives (FP). In addition, the number of true pixels (TP) was calculated by subtracting the total number of pixels identified by the algorithm from those deemed to be false positives. Finally, the number of true negative (TN) pixels was calculated by subtracting the total number of pixels in the image by the number of pixels identified by the algorithm, and by the number of false negatives.

Four different metrics were calculated to assess the performance of the algorithm. The sensitivity of the test evaluates the capability of the algorithm to identify pixels belonging to the tumour nests. The sensitivity was calculated as follows:

$$\text{Sensitivity}\left(\text{SE}\right)=\frac{\text{TP}}{\text{TP}+\text{FN}}\cdot \;100\%$$

The specificity of the test evaluates the capability of the algorithm to correctly identify the pixels not belonging to the tumour nests. The specificity was calculated as follows:

$$\text{Sensitivity}\left(\text{SP}\right)=\frac{\text{TN}}{\text{TN}+\text{FP}}\;\cdot \;100\%$$

The proportion of the histology slide occupied by the BCC may vary significantly between different slides. In general, superficial tumours occupy a smaller fraction of the slide compared to the nodular and infiltrative subtypes. For this reason the positive and negative predictive values were calculated. The positive predictive value (PPV) of the test indicates the probability that a positively identified pixel belongs to an actual tumour. As a result, images containing a lower tumour to non-tumour ratio result in lower PPVs. Conversely, the negative predictive value (NPV) is an indication of the probability of a negatively identified pixel actually belonging to non-tumour tissue. The PPV and NPV were calculated as follows:

$$\text{Positive}\;\text{Predictive}\;\text{Value}\;\left(\text{PPV}\right)=\frac{\text{TP}}{\text{TP}+\text{FP}}\;\cdot \;100\%$$

$$\text{Negative}\;\text{Predictive}\;\text{Value}\;\left(\text{NPV}\right)=\frac{\text{TN}}{\text{TN}+\text{FN}}\;\cdot \;100\%$$

## Results

**Figure 2:**
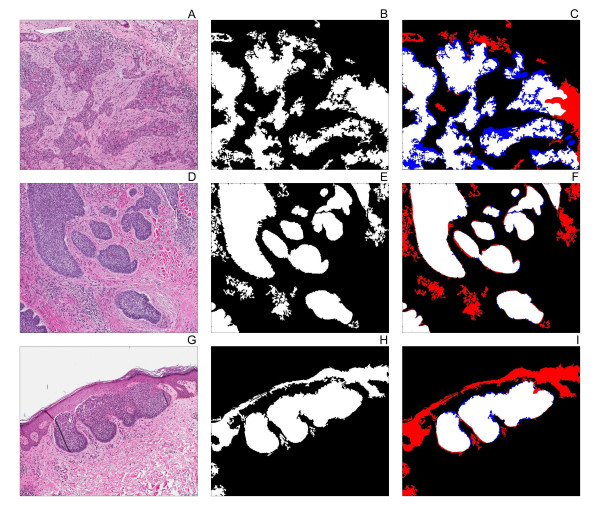
**Application of the tumour extraction algorithm to different basal cell carcinoma subtypes**. The algorithm was applied basal cell carcinoma images of the infiltrative (**A**), nodular (**D**), and superficial (**G**) subtypes. The white pixels correspond to the regions identified by the algorithm as tumour nests (**B, E, H**). The performance of the algorithm was evaluated by identifying pixels containing true positive pixels (white), false negative pixels (blue), and false positive pixels (red) in each image (**C, F, I)**.

The metrics shown in Table 1 quantify the performance of the algorithm. Overall, the mean sensitivity and specificity of the 30 test images was 91.0% and 86.4% respectively. Furthermore, the mean PPV and NPV of the test images was 63.3% and 94.2%. The algorithm performance depended significantly on the tumour subtype. We hypothesized that the algorithm would perform best on the superficial and nodular basal cell carcinoma subtypes, as these appear to be subjectively more "baseloid" than the infiltrative subtype. This was only partly supported by the data. The superficial subtype had the highest sensitivity (98.1%), but also the lowest specificity (82.5%). The lower specificity value resulted primarily from the extraction of the normal epidermal basal cell layer, in addition to the tumour nests. Consequently, this also resulted in a lower mean PPV (34.3%) for the superficial subtype. The infiltrative subtype had the lowest sensitivity (78.9%). On the other hand, the algorithm was more specific for the infiltrative subtype (87.2%). The algorithm performed the best with the Nodular subtype. The corresponding sensitivities and specificities were 95.8% and 89.3%, resulting in mean PPV and NPV values of 84.5% and 95.0%. A sample extraction of each subtype is shown in Figure 2.

**Table 1:** Evaluation of the tumour extraction algorithm in BCC histology slides

Tumours	n	Sensitivity (%)	Specificity (%)	PPV (%)	NPV (%)
Infiltrative	10	78.95	87.25	71.15	87.88

Superficial	10	98.13	82.50	34.34	99.74

Nodular	10	95.82	89.31	84.53	94.96

All	30	90.97	86.35	63.34	94.19

PPV: Positive Predictive Value = [True Positive Pixels/(True Positive Pixels + False Positive Pixels)] · 100%; NPV: Negative Predictive Value = [True Negative Pixels/(True Negative Pixels + False Negative Pixels)] · 100%; Sensitivity = [True Positive Pixels/(True Positive Pixels + False Negative Pixels)] · 100%; Specificity = [True Negative Pixels/(True negative Pixels + False Positive Pixels)] · 100%

**Figure 3:**
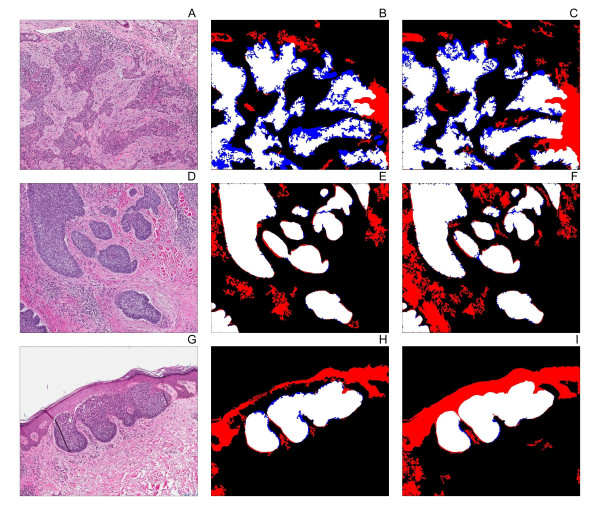
**Application of the tumour extraction algorithm with and without colour deconvolution**. The algorithm was applied basal cell carcinoma images of the infiltrative (**A**), nodular (**D**), and superficial (**G**) subtypes. The performance of the algorithm was evaluated by identifying pixels containing true positive pixels (white), false negative pixels (blue), and false positive pixels (red) in the images pre-processed using colour deconvolution (**B, E, H**), and the images without colour deconvolution (**C, F, I**).

Surprisingly, when compared to grayscale based segmentation (Table 2), the use of colour deconvolution resulted in a slightly lower mean sensitivity (91.0% with colour deconvolution; 91.6% with grayscale based segmentation), but increased the mean specificity (86.4% with colour deconvolution; 74.6% with grayscale based segmentation). The use of colour deconvolution prior to segmentation resulted in an improved PPV (63.3% with colour deconvolution; 52.6% with grayscale based segmentation) and NPV (94.2% with colour deconvolution; 93.9% with grayscale based segmentation). Sample extractions with and without colour deconvolution are shown in Figure 3.

**Table 2:** Evaluation of the tumour extraction algorithm without colour deconvolution in BCC histology slides

Tumours	Sensitivity (%)	Specificity (%)	PPV (%)	NPV (%)
Infiltrative	81.25	68.72	53.57	87.87

Superficial	98.46	74.20	27.34	99.78

Nodular	95.11	80.92	76.90	93.92

All	91.61	74.61	52.60	93.86

PPV: Positive Predictive Value = [True Positive Pixels/(True Positive Pixels + False Positive Pixels)] · 100%; NPV: Negative Predictive Value = [True Negative Pixels/(True Negative Pixels + False Negative Pixels)] · 100%; Sensitivity = [True Positive Pixels/(True Positive Pixels + False Negative Pixels)] · 100%; Specificity = [True Negative Pixels/(True negative Pixels + False Positive Pixels)] · 100%

## Discussion

This study evaluated a method for digitally extracting the tumour regions from basal cell carcinoma histopathology slides. A combination of colour deconvolution and intensity based thresholding was used with the goal of extracting the tumour nests from the image. The algorithm was evaluated with 3 separate subtypes of basal cell carcinomas: infiltrative, nodular, and superficial. For comparison, the algorithm was repeated using only grayscale based segmentation in place of the colour deconvolution step.

**Figure 4:**
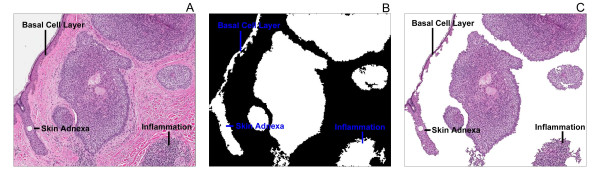
**False positive regions identified by the tumour extraction algorithm**. The basal cell layer, skin adnexa, and inflammation present in the original image (**A**) were identified as cancerous in the binary file (**B**), and the resulting extraction (**C**).

The performance of the algorithm varied significantly between the subtypes. The best results were achieved with the nodular subtype, while inferior performance was achieved with the superficial and infiltrative subtypes. One problem encountered with the superficial and infiltrative subtypes was the identification of false positives. This occurred in large part due to the presence of the normal epidermal basal layer in some of the slides which showed similar spectral characteristics to areas of BCC. Because the algorithm uses intensity based segmentation following the colour deconvolution step, regions with similar intensities to the tumour cells were also extracted. Lowering the threshold value would decrease the number of false positives, but would also come at the expense of lower sensitivity. Additional false positives occurred due to the presence of other basaloid elements such as skin adnexae, inflammation, and eccrine glands. A further source of false positives occurred due to blue dye used to mark the deep surgical resection margin of some specimens. Example false positives are displayed in Figure 4.

Another challenge for digital feature extraction algorithms is false negatives. In this study, false negatives resulted mainly from two causes: poor contrast between the tumour nest and its surrounding tissue, as well as inadequate hole filling. Although contrast enhancement was performed, some of the images still contained poor contrast between the tumour and its adjacent tissue. This may have been due in part to variation in the intensities of H&E staining of the original sections. One possible approach to this would be to explore the delineation of the tumour based on morphological features, rather than pixel intensities. One possibility would be to use the active contours method in order to evolve a curve representing the boundaries from the ROI [31]. Recently, this method has been explored in order to segment histology images [32-34]. One potential drawback when using active contours is that some implementations require the user to manually specify an initial boundary. Another possible approach would be to use region growing based segmentation [35]. This method works by adding pixels that surround, and are similar to a given seed pixel. The process is then repeated for each added pixel [18]. Similar to the active contours method, many region growing algorithms are not fully automated, as the given implementation may require the user's input to specify the seed for the algorithm. However, as we stated in the introduction, our intent was to examine the performance of a simple chromatin-rich segmentation algorithm and so these more complex approaches were not evaluated in the current study.

**Figure 5:**
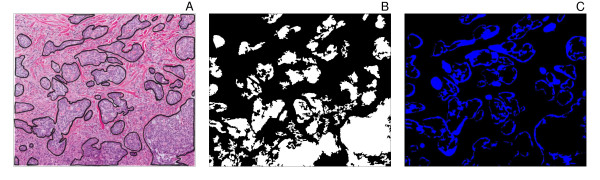
**False negative regions identified by the tumour extraction algorithm**. The ground truth dataset was determined manually (**A**). The white pixels correspond to the regions identified by the algorithm as tumour nests (**B**). The blue regions correspond to the false negative pixels identified by the algorithm (**C**).

Similar to the false positives, the rate of false negatives could be decreased by changing the threshold value. Conversely, lower false negative rates could be achieved at the expense of specificity. Example false negatives are shown in Figure 5.

Superior results were achieved by using a colour deconvolution prior to segmentation. Although using colour deconvolution resulted in a slightly lower mean sensitivity, a significant improvement in specificity was gained. This resulted in superior PPV and NPV values. In general, the colour deconvolution decreased the incidence of false positives. This was likely a result of the stain separation achieved using the colour deconvolution plugin.

Overall, however, the sensitivities of the colour-based approach were not better than a grayscale-based thresholding approach.

## Conclusions

This study reports the operational characteristics of a simple colour-based segmentation algorithm using the open-source image analysis program ImageJ. As predicted, the algorithm generally performed best with examples of the nodular basal cell carcinoma subtype. The specificity was unexpectedly low for the superficial basal cell carcinoma examples due to false positive classification of pixels associated with skin adnexae and the normal basal cell carcinoma of the epidermis. However, overall, the finding that the sensitivity of this colour-based approach was not better than a grayscale thresholding approach to the same images suggests that simple colour-based algorithms without the inclusion of more sophisticated texture feature segmentation may have limited utility.

## Competing interests

The authors declare that they have no competing interests.

## Authors' contributions

CN devised the original study design. KL designed the algorithm and evaluated the algorithm performance. Both authors participated in drafting the manuscript. Both authors have read and approved the final manuscript.

## Availability of supporting data

The ImageJ algorithm we used is available as an Additional file to this manuscript.

## Supplementary Material

Additional file 1 **ImageJ Algorithm**. Click here for file

## References

[B1] Pantanowitz L Digital images and the future of digital pathology J Pathol Infor 2010 1 15 10.4103/2153-3539.68332 PMC2941968 20922032

[B2] Gabril MY Yousef GM Informatics for practicing anatomical pathologists: marking a new era in pathology practice Mod Pathol 2010 23 349 358 10.1038/modpathol.2009.190 20081805

[B3] Dawson AE Can we change the way we screen?: the ThinPrep Imaging System Cancer 2004 102 340 4 10.1002/cncr.20721 15540250

[B4] Petushi S Garcia FU Haber MM Katsinis C Tozeren A Large-scale computations on histology images reveal grade-differentiating parameters for breast cancer BMC Med Imaging 2006 6 14 10.1186/1471-2342-6-14 17069651 PMC1634843

[B5] Karaçali B Tözeren A Automated detection of regions of interest for tissue microarray experiments: an image texture analysis BMC Med Imaging 2007 7 2 10.1186/1471-2342-7-2 17349041 PMC1838905

[B6] Hall BH Ianosi-Irimie M Javidian P Chen W Ganesan S Foran DJ Computer-assisted assessment of the human epidermal growth factor receptor 2 immunohistochemical assay in imaged histologic sections using a membrane isolation algorithm and quantitative analysis of positive controls BMC Med Imaging 2008 8 11 10.1186/1471-2342-8-11 18534031 PMC2447833

[B7] Safadi RA Musleh AS Al-Khateeb TH Al-Hadi Hamasha A Analysis of immunohistochemical expression of k19 in oral epithelial dysplasia and oral squamous cell carcinoma using color deconvolution-image analysis method Head and neck Pathol 2010 4 282 9 10.1007/s12105-010-0210-6 20882374 PMC2996498

[B8] LeAnder R Chindam P Das M Umbaugh SE Differentiation of melanoma from benign mimics using the relative-color method Ski Res Technol 2010 16 297 304 10.1111/j.1600-0846.2010.00429.x 20636998

[B9] Iyatomi H Oka H Celebi ME Hashimoto M Hagiwara M Tanaka M Ogawa K An improved Internet-based melanoma screening system with dermatologist-like tumor area extraction algorithm Comput Med Imaging and Graphics 2008 32 566 79 10.1016/j.compmedimag.2008.06.005 18703311

[B10] Abbas Q Celebi ME García IF Skin tumor area extraction using an improved dynamic programming approach Skin Res Technol 2011 11 1 10 10.1111/j.1600-0846.2011.00544.x 21507072

[B11] Silveira M Nascimento JC Marques JS Marcal ARS Mendonca T Yamauchi S Maeda J Rozeira J Comparison of segmentation methods for melanoma diagnosis in dermoscopy images IEEE J Sel Topics in Signal Process 2009 3 35 45

[B12] Miller SJ Biology of basal cell carcinoma (part I) J Am Acad Dermatol 1991 24 1 13 10.1016/0190-9622(91)70001-I 1999506

[B13] Miller DL Weinstock MA Nonmelanoma skin cancer in the United States: Incidence J Am Acad Dermatol 1994 30 774 778 10.1016/S0190-9622(08)81509-5 8176018

[B14] Gutiérrez R Gómez F Roa-Peña L Romero E A supervised visual model for finding regions of interest in basal cell carcinoma images Diagn Pathol 2011 6 26 10.1186/1746-1596-6-26 21447178 PMC3079595

[B15] Dougherty G Image segmentation Digital Image Process Med Appl 2009 1 Cambridge: Cambridge University Press 309 312

[B16] Russ JC Segmentation and thresholding The Image Processing Handbook 2002 4 Boca Raton: CRC Press 333 335

[B17] Dougherty G Image segmentation Digital Image Processing for Medical Applications 2009 1 Cambridge: Cambridge University Press 317 321

[B18] Dougherty G Image segmentation Digital Image Processing for Medical Applications 2009 1 Cambridge: Cambridge University Press 321 326

[B19] Dougherty G Image restoration Digital Image Processing for Medical Applications 2009 1 Cambridge: Cambridge University Press 52 253

[B20] Ruifrok AC Johnston DA Quantification of histochemical staining by color deconvolution Anal Quant Cytol Histol 2001 23 291 299 11531144

[B21] Konsti J Lundin M Joensuu H Lehtimäki T Sihto H Holli K Turpeenniemi-Hujanen T Kataja V Sailas L Isola J Lundin J Development and evaluation of a virtual microscopy application for automated assessment of Ki-67 expression in breast cancer BMC Clin Pathol 2011 11 3 10.1186/1472-6890-11-3 21262004 PMC3040126

[B22] Shah M Bhoumik A Goel V Dewing A Breitwieser W Kluger H Krajewski S Krajewska M DeHart J Lau E Kallenberg DM Jeong H Eroshkin A Bennett DC Chin L Bosenberg M Jones N Ronai ZA A Role for ATF2 in Regulating MITF and Melanoma Development PLoS Genet 2010 6 e1001258 10.1371/journal.pgen.1001258 21203491 PMC3009656

[B23] Wang CW Robust automated tumour segmentation on histological and immunohistochemical tissue images PLoS One 2011 6 e15818 10.1371/journal.pone.0015818 21386898 PMC3046129

[B24] Collins T ImageJ for Microsc BioTech 2007 43 S25 S30

[B25] Abràmoff MD Magalhaes P Ram S Image processing with ImageJ Biophotonics Int 2004 11 36 43

[B26] Landini G Colour deconvolution plugin v 1.5 http://www.dentistry.bham.ac.uk/landinig/software/cdeconv/cdeconv.html

[B27] The ImageJ information and documentation portal http://imagejdocu.tudor.lu/doku.php?id=faq:technical:what_is_the_algorithm_used_in_automatic_thresholding

[B28] Zhang H Fritts J Goldman S Image segmentation evaluation: A survey of unsupervised methods Comput Vision and Image Understanding 2008 110 260 280 10.1016/j.cviu.2007.08.003

[B29] Cardoso JS Corte-Real L Toward a generic evaluation of image segmentation IEEE Trans Image Process 2005 14 1773 82 16279178 10.1109/tip.2005.854491

[B30] Udupa JK Leblanc VR Zhuge Y Imielinska C Schmidt H Currie LM Hirsch BE Woodburn J A framework for evaluating image segmentation algorithms Comput Med Imaging and Graphics 2006 30 75 87 10.1016/j.compmedimag.2005.12.001 16584976

[B31] Kass M Witkin A Terzopoulos D Snakes: Active contour models Int J Comput Vis 1988 1 321 331 10.1007/BF00133570

[B32] Xu J Janowczyk A Chandran S Madabhushi A A high-throughput active contour scheme for segmentation of histopathological imagery Med Image Anal 2011 15 851 62 10.1016/j.media.2011.04.002 21570336 PMC3168681

[B33] Fatakdawala H Xu J Basavanhally A Bhanot G Ganesan S Feldman M Tomaszewski JE Madabhushi A Expectation-maximization-driven geodesic active contour with overlap resolution (EMaGACOR): Application to lymphocyte segmentation on breast cancer histopathology IEEE Trans Biomed Eng 2010 57 1676 89 20172780 10.1109/TBME.2010.2041232

[B34] Hiremath PS Iranna YH Fuzzy rule based classification of microscopic images of squamous cell carcinoma of esophagus Int J Comput Appl 2011 25 30 33

[B35] Mat-Isa N Mashor M Othman N Seeded region growing features extraction algorithm; its potential use in improving screening for cervical cancer Int J Comput Internet and Manage 2005 13 61 70

