# Supplementary material for: Performance of a simple chromatin-rich segmentation algorithm in quantifying basal cell carcinoma from histology images
Source: BMC Res Notes. 2012 Jan 17;5:35. doi: 10.1186/1756-0500-5-35 (PMC3398325; doi:10.1186/1756-0500-5-35)
Supplement: Additional file 1 — ImageJ Algorithm. [file 1756-0500-5-35-S1.DOC]

**Supplementary Data File: ImageJ Algorithm**

// Obtains the title of the open windows. Used to select the desired window.

if (nImages>0)

{

title = getTitle();

//title

print("title: " + title);

jpeg_ext =".jpg";

jpeg_pos= indexOf(title,jpeg_ext,0);

title = substring(title,0,jpeg_pos);

print("title: " + title);

}

// Runs the colour deconvolution plugin with the H&E preset

run("Colour Deconvolution", "vectors=H&E");

windowTitle= title+".jpg-(Colour_3)";

selectWindow(windowTitle);

//Closes unused windows

close();

close();

// Selects the desired window

selectWindow("Colour Deconvolution");

close();

//Enhances contrast, thresholding, creates a binary image, and performs morphological operations

run("Enhance Contrast", "saturated=0.4 equalize");

setAutoThreshold("Default");

run("Threshold...");

run("Make Binary", "thresholded remaining black");

run("Remove Outliers...", "radius=2 threshold=50 which=Bright");

run("Fill Holes");

run("Remove Outliers...", "radius=2 threshold=50 which=Dark");

// Obtains particles >= 750 pixels

run("Analyze Particles...", "size=750-infinity circularity=0.00-1.00 show=Nothing include add");

//close();
